# Contact dermatitis caused by prevention measures during the COVID-19 pandemic: a narrative review

**DOI:** 10.3389/fpubh.2023.1189190

**Published:** 2023-07-20

**Authors:** Huimiao Tang, Hao Wang, Michael R. Hamblin, Lu Jiang, Yanjun Zhou, Yidan Xu, Xiang Wen

**Affiliations:** ^1^Department of Dermatology, West China Hospital, Sichuan University, Chengdu, China; ^2^Laboratory of Dermatology, Clinical Institute of Inflammation and Immunology, Frontiers Science Center for Disease-related Molecular Network, West China Hospital, Sichuan University, Chengdu, China; ^3^Laser Research Centre, Faculty of Health Science, University of Johannesburg, Doornfontein, South Africa; ^4^Outpatient Department of West China Hospital, Sichuan University, Chengdu, China

**Keywords:** COVID-19, contact dermatitis, personal protective equipment, dermatology, hygiene

## Abstract

**Introduction:**

During the outbreak of Coronavirus disease 2019 (COVID-19), health care workers wore personal protective equipment including masks, gloves and goggles for a long time. In order to reduce the transmission routes of the virus, public places were sprayed with disinfectant. Moreover, the body, hands and clothing were frequently disinfected and washed for hygiene purposes. Studies have shown that these practices could easily irritate the skin and damage the skin barrier. Long-term irritation or exposure to allergens may lead to the occurrence of contact dermatitis (CD).

**Methods:**

Subject headings were searched via the National Library of Medicine (PubMed) and web of science databases: COVID-19; contact dermatitis; adverse skin reaction; PPE; dermatitis; mask; glory; hand hygiene, disinfection; face shield; goggle; protect cloth. A total of 246 and 646 articles were retrieved from the two databases, respectively. 402 articles remained after removing duplicates. Reviews, non-English articles, articles that could not be accessed to read or did not conform to our topic were excluded. Finally, a total of 32 cross-sectional studies, 9 case reports and 2 randomized controlled trials were included.

**Discussion:**

This article reviews reports of CD caused by various prevention and hygiene measures during the COVID-19 pandemic. The amount of skin damage caused by COVID-19 prevention measures could be decreased by improved education about skin management.

## 1. Introduction

In December 2019，the novel Coronavirus disease 2019 (COVID-19) caused by the SARS-CoV-2 virus first became known to the public and quickly rose to pandemic status all over the world (1, 2). According to the World Health Organization, up to 30 January 2023, the number of people diagnosed with COVID-19 had reached 753,001,888 with 6,807,572 deaths reported (3). The advent of COVID-19 vaccines has dramatically reduced the number of infections in the pandemic, but the emergence of new virus variants has made prevention and control difficult (4). In the last 3 years, to combat the overwhelming COVID-19 pandemic, countless health care workers (HCWs) strived on the front line to protect the health and life of the general public. Considering the origin and various modes of SARS-CoV-2 transmission (5, 6), effective preventive measures have been shown to play a great role in reducing the risk of infection (7). Personal protective equipment (PPE) including gloves, masks, N95 respirators, goggles, face shields and gowns) was widely employed. Moreover, other preventive measures (hand sanitizer and disinfectants) were used by HCWs, while preventive measures and masks were also recommended for non-HCWs (8).

However, the long-time wearing of PPE and frequent hand washing or disinfection could often lead to adverse skin reactions (ASR), such as contact dermatitis (CD), as one of the most common dermatoses (9, 10). CD is an inflammatory reaction in which the skin at the contact site becomes inflamed due to exposure to exogenous substances, it can be divided into irritant contact dermatitis (ICD) and allergic contact dermatitis (ACD) according to the etiology. 80% of CD cases were classified as ICD. ICD is caused by exposure of the skin to the irritants, resulting in changes in the epidermal barrier, with itching, pain, burning as the main symptoms. ACD is mainly caused by the activation of cell-mediated immune response by chemical lipophilic molecules, which is mainly manifested as pruritus (11). In a recent cross-sectional study, 65.3% of participant HCWs were self-diagnosed with skin lesions, 25.8% of which were contact/atopic dermatitis (10). One study reported that during the pandemic, CD accounted for 16.5% of PPE-related occupational skin disorders. The most affected parts of the body were the bridge of the nose (24.7%), cheeks (21.3%), forehead (10.3%) and palms (2.8%) (12). Montero-Vilchez et al. reported that CD was the most common ASR associated with PPE (13). Therefore, this review focuses on the development CD as a result of exposure to COVID-19 prevention measures.

Wearing PPE for more than 6 h per day increases the chances of developing ASRs (14). Eczema, pruritus, erythema, edema, urticaria-like plaques, blisters, erosion, exudative lesions, scaling and desquamation are the main manifestations of CD. CD can not only cause health issues for HCWs in the process of performing their medical duties, but can also affect non-HCWs exposed to preventive measures like masks and disinfectants.

## 2. Contact dermatitis caused by PPE during the COVID-19 pandemic

### 2.1. Contact dermatitis caused by mask wearing

**Figure 1:**
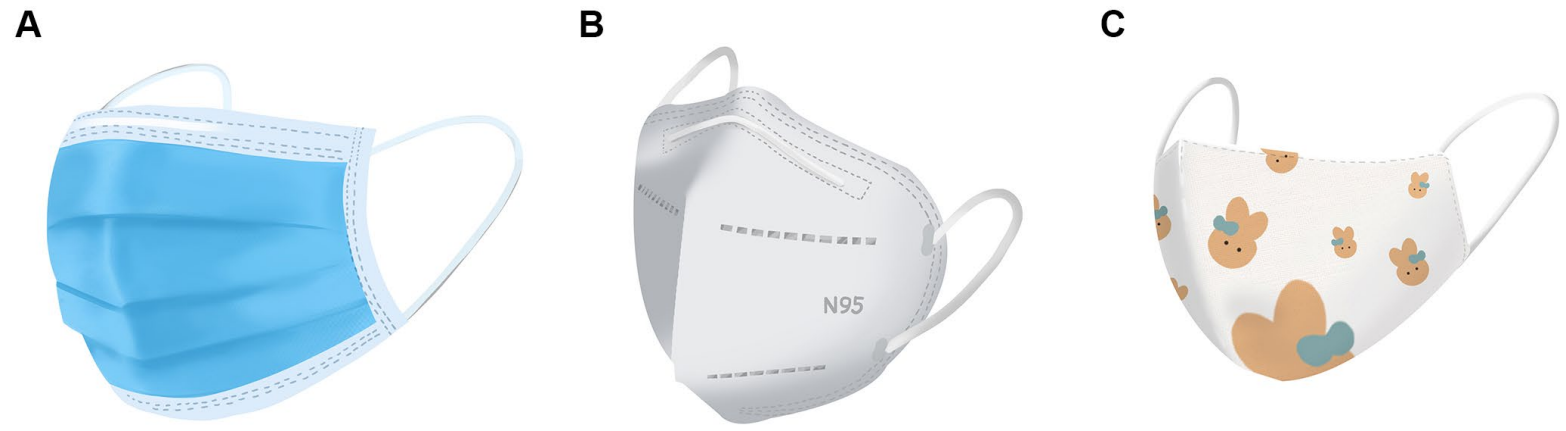
Various masks commonly used during the COVID-19 pandemic **(A)** surgical mask; **(B)** N95 mask; **(C)** cloth mask.

Because SARS-CoV-2 is mainly transmitted by air-borne droplets and according to the previous clinical experience obtained with protection against severe acute respiratory syndrome (SARS), masks were recommended as crucial for preventing the virus from being spread. Masks became daily necessities for HCWs involved with COVID-19 prevention and treatment, as well as being widely used by the general public (5, 15). Research has shown that wearing a mask can greatly cut down the risk of contracting COVID-19 (16). Figure 1 shows several commonly used types of masks. In a cross-sectional study, 37.8% (73/193) of HCWs wore a mask for more than 8 h a day, as compared to 16.5% (313/1897) of non-HCWs. The prolonged skin contact with the mask could cause various ASRs, such as redness (51%), pruritus (49.5%) and acne (43.7%) and of these, 6.2% of HCWs were diagnosed with CD (17). The bridge of the nose, ears, cheeks, perioral area and the chin were the most common sites to be affected (18).

Long-term wearing of masks may cause an increase in the surface temperature of the covered skin (19). The warm, humid and closed environment is beneficial to the growth of microorganisms, while the secretion of pilosebaceous glands can be blocked, resulting in the development of acne. Excessive compression may lead to the occurrence of skin pressure ulcers (20). The occlusive environment makes the skin more permeable and more sensitive to irritation by physical friction/pressure or by exogenous chemicals, eventually leading to CD (21).

In a previous study in the Philippines, the prevalence of mask-induced CD was 34.6%, the variation may due to a large difference in the distribution of HCWs and non-HCWs (22). Skiveren et al. (23) found that the prevalence of ASRs due to face-masks in HCWs was 61.9% using an online questionnaire. Cazzaniga et al. (24) showed that about 18.4% of mask wearers in the community experienced ASRs, including redness, itching, swelling and erosion in the mask contact area.

### 2.2. Surgical masks

Surgical masks are the predominant type worn by non-HCWs, due to their safety and relative availability. Wear surgical masks correctly can greatly lower the emission of particles associated with aerosol-generating procedures (AGP) by 90% when speaking or coughing. These masks could effectively reduce the number of particles of influenza virus released from the respiratory tract into the environment (25, 26). Nevertheless, the longer the mask is worn even if replaced, the greater the risk of CD (27).

A typical surgical mask usually includes three or four layers. The inner layer is a soft absorbent sheet, the middle one or two layers are polypropylene barriers and the outer layer is melt-blown non-woven fabric with water resistance (28). The main facial lesions caused by surgical masks were redness (55/142), itching (49/142), dryness (20/142), acne (10/142) and rash (8/142) (29). Long-term wearing of polypropylene surgical masks may lead to ACD because formaldehyde, a known ACD sensitizer, is a decomposition product of polypropylene (30, 31). Coco-propylenediamine-guanidinium diacetate and dibromodicyanobutane, which are used in surgical masks are also potential allergens (32). In addition, the elastic bands on surgical masks can also contribute to ACD. The chemical promoters used to accelerate the vulcanization of rubber (including antioxidants) are allergens contained in the elastic bands of surgical masks (33). It is worth noting that although the metal strips (containing nickel and cobalt) on the nose bridge of the masks do not directly contact the skin, they could still contribute to ACD (34). Table 1 shows the main materials, potential allergens, lesion sites and manifestations of CD caused by various types of PPE.

**Table 1:** The main materials, potential allergens, sites of skin lesions and symptoms caused by PPE.

PPE		Material	Allergen	Body regions	ASR
Masks	Surgical mask	Soft absorbent sheets, Polypropylene barriers, Melt-blown non-woven fabric	Formaldehyde, 2-bromo-2-nitropropane-1,3-diol, Coco-propylenediamine-guanidinium diacetate, Dibromodicyanobutane Vulcanization promoters, Antioxidants, Nickel, Cobalt	Nasal, Bridge, Ears, Cheeks, Perioral, Chin	Redness, Itching, Dryness
	N95/KN95 respirator	Skin-friendly layer, Structural support filter layer, Hydrophobic coating layer	Formaldehyde, Thiurams, Dithiocarbamates, Mercaptobenzothiazole		Redness, Itching
	Cloth mask	Cotton, Polyester	Formaldehyde, Formaldehyde textile resins, Formaldehyde releasers, Disperse dyes, P-aminobenzene, P-phenylenediamine		Erythema, Scaling
Gloves		Latex, Nitrile rubber, Plastic	Latex, Carba mix, Mercaptobenzothiazole (MBT), Thiuram mix	Hands	Dryness, Rash, Itching
Protective clothing		Polypropylene melt-blown cloth, Polyester fiber	Vinyl, Rubber materials	Limbs, Trunk	Dryness, Pruritus
Protective goggles		Polycarbonate, Optical resin, Polymethyl methacrylate	Not available at present	Nasal bridge	Pressure, Sores, Rash
Face shields		Elastic, Headband, Polycarbonate	Not available at present	Forehead	Abrasions, Itching

Aerts et al. reported a case of ACD due to a polypropylene surgical mask containing formaldehyde and 2-bromo-2-nitropropane-1,3-diol, along with with recurrent rosacea (35). A systematic review presented three cases of CD caused by surgical masks, with symptoms including erythema, itching and burning sensation on the face (36). In another study, one case of ACD and two cases of ICD (both associated with double-layer surgical masks) were reported with erythema and scaling involving the retroauricular area (37).

### 2.3. N95/KN95 respirators

N95/KN95 respirators filter out 95% of particles and are more effective than surgical masks in preventing emission of particles from AGPs (sneezing and coughing), making them a good choice for HCWs (28, 29, 38–40). They consist of an inner skin-friendly layer, two structural support filter layers in the middle (mainly made of polyethylene) and an outer hydrophobic coating layer (mostly made of polypropylene) (28, 31). However, because the N95/KN95 respirator fits tighter on the face, it is responsible for more ASRs compared to surgical masks (24). Formaldehyde has been detected in N95/KN95 respirators, accelerating the possible development of ACD (30, 41, 42). In addition, the elastic bands of the N95 respirator along with the sponge strip in the masks have been reported to contribute to the occurrence of ACD. The culprit agents were proposed to be rubber additives in the elastic bands (such as thiurams, dithiocarbamates, or mercaptobenzothiazole), or polyurethane sponge in the respirator (30, 43, 44). The N95/KN95 respirators are attached by ear bands which can cause physical pressure ulcers on the ears (45). Postauricular dermatitis is another form of CD that sometimes occurs after wearing N95/KN95 respirators. Bothra et al. (37) reported four occurrences of ACD (2 cases) or ICD (2 cases) after N95/KN95 respirator use, with symptoms including erythema, desquamation and papules. Most HCWs wear a N95/KN95 respirator for a long time, so the incidence of facial dermatitis could be as high as 81.69%, which mainly consists of redness and itching of the nose and cheeks (29).

Different masks can be selected by the public according to different protection needs. HCWs who caring for COVID-19 patients are recommended to use N95/KN95 respirators, this kind of occupational injury is sometimes unavoidable. In order to reduce skin damage, ordinary surgical masks are the appropriate choice for general people who do not go to public venues or contact with COVID-19 patients in hospitals.

### 2.4. Cloth masks

Cloth masks are not as efficient as surgical masks or N95 respirators in filtering particulate matter, but have been used during the COVID-19 pandemic when mask supplies were scarce (24, 46–48). In one study, free formaldehyde was found in cotton masks or polyester masks, which may cause ACD (49). In addition, textiles can contain formaldehyde resins or can release formaldehyde, disperse dyes, p-aminobenzene and p-phenylenediamine, which are all potential sensitizing factors (32). However, compared with surgical masks and N95 respirators, cloth masks may cause fewer ASRs (50). In contrast, another study showed no significant difference in skin reactions between cotton masks and surgical masks (51). Bothra reported one case of ACD characterized by erythema and scaling caused by the use of a cloth mask (37). Therefore, although the prevalence rate is low, long-term cloth mask wearers should not ignore the possibility of CD.

To prevent mask-induced CD the following measures can be taken, thin hydrocolloid dressings or thin foam dressings can be applied prophylactically, washing of the face with a mild, scented cleanser at morning and night, wear a head-attached mask instead of an ear-attached mask and take a break every 2 hours after wearing a mask (52).

### 2.5. Contact dermatitis caused by gloves

**Figure 2:**
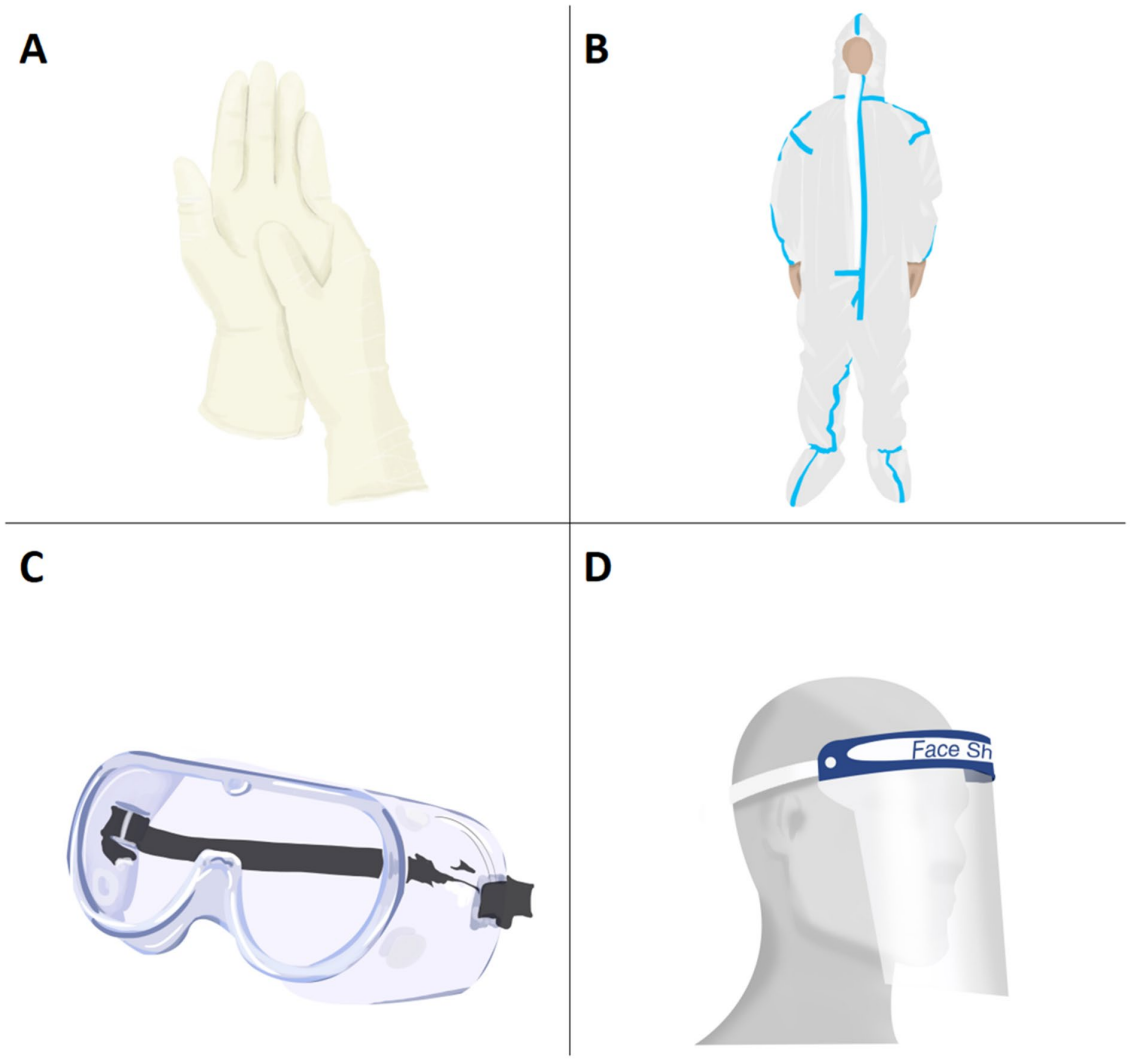
Various PPE in addition to masks **(A)** glove; **(B)** protective clothing; **(C)** protective goggle; **(D)** face shield.

During the COVID-19 pandemic, hand to mouth contact is another major transmission route of SARS-CoV-2, so gloves are essential for medical staff (5). As the duration of glove wearing increased, it was found that the risk of ASR also increased (53). HCWs with skin lesions wore PPE for more than 8 h per day on average (54). Figure 2 shows several types of PPE in addition to masks. In order to increase their own safety, some HCWs choose to wear multi-layered gloves. One survey showed that 25.7% of HCWs liked to wear double-layer gloves, among which 62% had complained of hand skin irritation (55). In another survey, among all participants with hand skin lesions, 69.9% of HCWs wore double gloves and 24.3% wore triple gloves (56). However, there is no evidence that increasing the number of glove layers worn could provide better protection against COVID-19 infection. On the contrary, multiple layer gloves actually increased the likelihood of hand CD (21). Therefore, it is recommended to reduce the number of layers of latex gloves consistent with appropriate and safe protection, so as to reduce the risk of hand skin damage. If wearers are allergic to latex, cotton or plastic gloves can be worn inside the latex gloves, while patch testing for specific allergens is needed to know which materials to avoid.

The components of medical gloves are variable. Due to their strong durability, latex and nitrile rubber are often the preferred materials for gloves, while plastic polyvinylchloride (PVC) gloves, have also won the favor of some HCWs, because of their hypoallergenic properties. However, additives, such as carba mix (diphenylguanidine, zinc-dibutyldithiocarbamate and zinc-diethyldithiocarbamate), mercaptobenzothiazole (MBT), or thiuram mix (tetramethylthiuram monosulfide, disulfiram, tetramethylthiuram disulfide and dipentamethylenethiuram disulfide) which could be contained in PVC could also contribute to ACD (57). Alves et al. (58) reported one case of ACD caused by an allergy to MBT during the COVID-19 pandemic. Several hours after wearing latex gloves, vesicular erythema with itching developed on the hands and wrists. The powder in gloves may promote the development of hand itching and eczema, so powder free gloves could be an option for some individuals, while hands should be dried after washing to reduce the risk of maceration (59).

One study found that among the complications caused by glove wearing, dryness (75%) and rash/itching (72.2%) were the main complaints (60), which was consistent with previous studies by Sliva et al. (61) and Xia et al. (62). In addition, sweating and redness could be also hand symptoms (63). In another study of ASRs related to the use of latex gloves, after using latex gloves for an average of 10 h per day for about 3.5 months, 54 (88.5%) of the surveyed HCWs, complained of some dermatitis symptoms, such as dry skin (55.7%), itching (31.2%), rash (23.0%) and chapped skin (21.3%) (64).

Overhydration of the stratum corneum is a possible consequence of prolonged glove wearing, further accelerating the development of maceration and erosion, the chemical materials in latex glove may contribute to the development of CD (21). Hand cream can improve the maceration, but if irreversible erosion and exudation occurs, 3% boric acid solution aqueous dressings or topical application of zinc oxide ointment may be necessary. In patients with CD, topical glucocorticoids can be used (21).

### 2.6. Contact dermatitis caused by protective clothing

Protective clothing is generally used by HCWs in high-risk areas, but long working hours, (especially in areas with high ambient temperatures in summer) will undoubtedly cause some discomfort for HCWs (65). This could promote long-term contact of the skin with sweat and heat, which could eventually become a causative factor of CD (66, 67).

The incidence of occupational CD involving protective clothing is rare, with only 143 (3.6%) reported in a recent global systematic review with a total cohort size of 3,958 individuals (12). In a previous study by Hu et al. (64) the occurence of ASRs due to protective clothing was 60.7%, with dry skin (36.1%) and pruritus (34.4%) being the most common complaints. Among the dermatoses associated with the use of protective clothing, ICD stands out (61), with itching and rash the main symptoms caused by prolonged close friction and pressure irritation (60). In another study, ASRs were reported by 11% of 175 HCWs who regularly wore protective clothing, with the most common symptoms being pruritus and erythema and one case of rash (68). These numbers were quite different from the sturdy by Hu et al. (64). The reason may be that the Hu et al. study took place early in the outbreak of COVID-19. The increase in the number of cases has contributed to work intensity and pressure on medical staff. In high pressure situations, HCWs tend to wear protective clothing for longer and have less chance to take it off.

To reduce the incidence of ICD caused by protective clothing, moisturizers or emollients can be used in the pressure areas on the body (69). If conditions permit, HCWs can control the wearing time and regularly remove PPE to prevent or mitigate excessive skin temperatures and sweating.

### 2.7. Contact dermatitis caused by protective goggles

The use of goggles can effectively prevent SARS-CoV-2 from entering the eyes through small droplets (70, 71). During the COVID-19 pandemic, 67% of HCWs used goggles for more than 4 h a day (18). The most common complications were physical stress-related skin lesions and CD may result from damage to skin integrity caused by prolonged mechanical friction (21, 60). The skin lesions may progress from erythema and depression to erosion and ulceration (12). Pressure sores and rashes are the most common dermatoses caused by goggles (61). Irritation by excessive or prolonged sweating can act as an accelerator (54).

One survey showed that 58% of skin problems were related to goggles (68), which agreed with the previous findings of Lan J et al. and the bridge of the nose was the most susceptible area after wearing goggles for more than 6 h per day (72). Goggle wearing could be a cause of ICD, so in order to reduce the skin pressure injury around the eyes and nasal bridge of HCWs, they are advised to take them off regularly, wipe off sweat, avoid using latex bands that can cause allergies and apply skin cream before wearing (54).

### 2.8. Contact dermatitis caused by face shields

The full-length PPE face mask typically consists of an elastic headband and a clear polycarbonate, sheet to protect the face from direct contact with aerosols or fluid splashes (73). It carries the risk of causing pressure sores and rashes (61). Abrasions, itching, pain and other changes in skin properties may also occur as a result of prolonged wearing of face shields (74). One study showed that of all the skin problems caused by PPE, face shields accounted for 23% and the forehead was the most affected site (68). In a previous study, the most common skin problems encountered in the management of COVID-19 was CD and 17.31% of these cases were caused by face shields of all types of PPE (54).

## 3. Contact dermatitis caused by disinfectant products during the COVID-19 pandemic

### 3.1. Contact dermatitis caused by hand hygiene products

Hand hygiene is particularly important in order to protect individuals from being infected during contact with infected people or in public places. The WHO and the China CDC recommend using alcohol-based hand sanitizer (ABHS), regular soap, or alcohol-free hand sanitizer (AFHS) to combat COVID-19 transmission (75, 76). The main ingredients of ABHS formulations are, ethanol, isopropanol, hydrogen peroxide, glycerol and water. The alcohol concentration ranges between 60 and 95% as the standard for optimal bactericidal and virucidal activity (77). The active ingredients of AFHS are quaternary ammonium compounds, iodophor and chloride. In addition, additives such as excipients and preservatives are commonly used in hand sanitizers (78). Soap contains surfactants, moisturizers, emulsifiers, perfume and various additives that are used to lower the risk of viral transmission (79). Table 2 lists the active ingredients, allergenic substances and resulting ASRs of some hand hygiene products.

**Table 2:** Active ingredients, allergens and hand ASRs of different hand hygiene products.

Hand hygiene products	Dominant sector	Sensitizer	ASR
ABHS	Ethanol, Isopropanol, Hydrogen, Peroxide, Glycerol	Ethanol, Isopropanol, Preservative agent, Quaternary ammonium chloride, Chlorhexidine, Triclosan, Chlorocresol, Phenoxyethanol, Myristool, Benzalkonium chloride	Dryness, Peeling, Itching
AFHS	Chlorides, Iodides, Peroxides, Phenols, Biguanide	Benzalkonium chloride, Cetroamide, Chlorocresol, Chlorhexidine, Triclosan, Sodium hypochlorite, Povidone-iodine	Dryness, Peeling, Skin color change
Soap	Surfactant, Emulsifier, Moisturizer, Fragrance, Coloring agent	Spice, Tocopherol, Polyethylene glycol, Ethylhexyl glycerol, Quaternary ammonium salts, Sezolinone, Sodium benzoate, Phenoxyethanol, Chlorocresol, Polyethylene glycol, Triclosan, Chlorhexidine gluconate, Iodophor, Povidone iodine	Dryness, Peeling, Eczema

However, frequent hand disinfection with alcohol and hand washing with soap or hand sanitizer during the COVID-19 pandemic can result in the development of ICD and ACD (80–82). Various chemical additives (including disinfectants and fragrances) present in hand hygiene products may be responsible for the increase of hand ACD. Typical allergens or irritants include ethanol, isopropanol, perfumes, quaternary ammonium salts, iodine, chlorhexidine, triclosan, chlorocresol, sodium benzoate, phenoxylethanol and stearols (32). Isopropanol disrupts the lipid bilayer structure between cells, leading to denaturation of proteins (83). Triclosan, chlorhexidine and quaternary ammonium compounds have been suggested to contribute to the development of dermatitis (79, 84).

The surfactants contained in hand hygiene products can remove the natural oils of the hands, disrupt the skin barrier and frequent exposure of the hands to water can also lead to increased skin permeability and separation of the stratum corneum, enhancing the irritating effects of surfactants on the skin and leading to ICD (74, 85–87).

It is clear that hand-washing for greater than 10 s 8–10 times per day using hand hygiene products significantly raises the risk of eczema and dryness on the hands (88, 89). Hand hygiene and disinfection can also exacerbate existing eczema and induce new skin problems (90). Most HCWs washed their hands more than 10 times a day for an average of 20 s while managing the COVID-19 pandemic (18, 72, 91). Hand skin damage is also common for workers in the general public who need to maintain regular hand hygiene (92). A cross-sectional study in Bangladesh showed that 41.8% of participants experienced ASRs due to long-term use of hand cleaning products. Most people used ABHS (75.53%) and/or soapy water (69.35%), while fewer used only AFHS (1.22%). Dry skin (34.39%) and peeling (11.71%) were the most common symptoms of ASRs. ABHS users were more easily to experience pruritus (8.13%), while soapy water users were more easily to experience peeling (12.9%), rashes (7.46%) and AFSH users were more easily to experience skin color changes (13.33%) (9). These numbers were analogical to the findings reported by Dash et al. (93), Abdi et al. (29) and Cebeci et al. (94).

Of the body parts affected by wearing PPE, hands are the most common, yet in practice only a few people use hand creams or moisturizers (94, 95). For prevention, rinsing of hands with warm water, the use of moisturizers or hand creams can replenish the moisture and lipids on the skin surface, restoring the skin barrier (79). While ABHS can also dissolve natural lipids in the epidermis, studies have found that ABHS are less damaging to the epidermal barrier compared to soap (96). Therefore, individuals who are not allergic to alcohol are advised to choose ABHS instead of soap. To reduce the occurrence of allergies, the choice of non-perfumed hand sanitizers and soaps is also recommended.

### 3.2. Contact dermatitis caused by environmental disinfectants

In some countries, coastal areas and densely populated cities have a higher risk of virus transmission (97). Recognizing the potential risk of airborne transmission of SARS-CoV-2, the use of environmental disinfection can help reduce any residual virus on indoor surfaces and in the air, especially in hospitals, classrooms, shopping malls and other places where crowds gather (98, 99). Sodium hypochlorite, ethanol, isopropanol and glutaraldehyde are examples of environmental disinfectants in common use (100). Extensive spraying of disinfectants throughout the environment may cause disinfectants to remain on the skin of people who come into contact with surfaces. Because many disinfectants are fat soluble, they can penetrate the surface of the skin, leading to the occurrence of ASRs. For example, alcohol causes dryness/itching/burning, chloride causes burning/pain/redness/blisters, aldehydes cause yellow-brown discoloration, while disinfectants are regarded as potential causes of for irritations and allergic skin diseases (101, 102). In a survey of household disinfectant use, ASRs occurred in 8% of respondents who used cleansers or disinfectants (103). Avoiding the overuse of excessive concentrations of environmental disinfectants is probably worth considering.

### 3.3. Contact dermatitis caused by disinfectants for clothing

Common disinfectants for clothing include phenolic compounds, quaternary ammonium salts and chlorine-producing disinfectants (104). The occurrence of the COVID-19 increased the frequency of disinfectant use in laundry and clothes washing. Benzalkonium chloride, a kind of quaternary ammonium cationic detergent was recommended in the cleaners for clothing of AIDS patients, but has been reported several times to cause ACD and children were even more susceptible (84, 105–108). Children who wore clothing treated with benzalkonium chloride in the laundry process, experienced symptoms such as erythema, tenderness, itching, rash and scaling (106). However, CD caused by clothing disinfection is still rare and understanding its mechanism of self-sensitization is the way to prevent it.

## 4. Conclusion

For the purpose of dealing with the overwhelming COVID-19 pandemic, everyone especially medical workers, have been faced with great challenges. PPE has become a weapon in the fight against the virus, while hand cleansers and disinfectants used appropriately are also important to reduce the transmission routes of this disease. However, the skin damage caused by COVID preventative measures deserves some attention. Many studies have reported the skin damage caused by it and CD is one of the common diseases. In order to have a comprehensive understanding of CD caused by protective measures, the review discussed the CD caused by masks, disinfectants and other PPE to the general public, especially HCWs during the pandemic. Involving a relatively complete set of protective equipment. Masks, gloves, protective clothing, goggles, face masks and disinfectants that may appear in various occasions are comprehensively summarized. The possible skin lesions were discussed, the components of various protective equipment and the potential allergens were also listed.

In particular, the CD related damage to the skin caused by environmental and clothing disinfectants mentioned in this article tends to be ignored in the previous study. The public needs to be educated about the choice of appropriate prevention measures and avoid skin damage with excessive protection. Moisturizers and mild skin care products can effectively reduce skin damage and prevent the occurrence of CD. At the same time, attention should be paid to reducing the frequency and duration of PPE wearing, especially when epidemiologically unnecessary. In cases of serious skin damage, it is recommended to seek the help of dermatologists. This review aims to summarize the contact dermatitis caused by various protective measures against COVID-19 and makes targeted recommendations on ways to reduce the associated lesions, we also propose future research directions for how to decrease the occurrence of skin lesions in the face of a pandemic with effective protection.

## Author contributions

HT and HW performed the literature search and wrote the first draft of the article. MH, LJ, YZ, and YX critically revised the manuscript for content and meaning. XW had the original idea for the article and critically revised the work. All authors contributed to the article and approved the submitted version.

## Funding

This work was supported by National Natural Science Foundation of China (no. 81903226).

## Conflict of interest

The authors declare that the research was conducted in the absence of any commercial or financial relationships that could be construed as a potential conflict of interest.

## Publisher’s note

All claims expressed in this article are solely those of the authors and do not necessarily represent those of their affiliated organizations, or those of the publisher, the editors and the reviewers. Any product that may be evaluated in this article, or claim that may be made by its manufacturer, is not guaranteed or endorsed by the publisher.
